# Effects of cRG-I Prebiotic Treatment on Gut Microbiota Composition and Metabolic Activity in Dogs In Vitro

**DOI:** 10.3390/microorganisms13081825

**Published:** 2025-08-05

**Authors:** Sue McKay, Helen Churchill, Matthew R. Hayward, Brian A. Klein, Lieven Van Meulebroek, Jonas Ghyselinck, Massimo Marzorati

**Affiliations:** 1 NutriLeads BV, Bronland 12N, 6708 WH Wageningen, The Netherlands; 2 dsm-firmenich, 60 Westview Street, Lexington, MA 02421, USAbrian.klein@dsm-firmenich.com (B.A.K.); 3 ProDigest BV, Technologiepark 82, 9052 Zwijnaarde, Belgium; lieven.vanmeulebroek@prodigest.eu (L.V.M.); jonas.ghyselinck@prodigest.eu (J.G.); massimo.marzorati@prodigest.eu (M.M.); 4 Center for Microbial Ecology and Technology (CMET), University of Ghent, Coupure Links 653, 9000 Gent, Belgium

**Keywords:** canine gut microbiome, carrot rhamnogalacturonan-I, cRG-I, dietary fiber, metagenomics, prebiotic

## Abstract

Low-dose carrot rhamnogalacturonan-I (cRG-I) has shown consistent modulatory effects on the gut microbiota and immune function in humans. In this study we investigated its effects on the microbial composition and metabolite production of the gut microbiota of small (5–10 kg), medium-sized (10–27 kg), and large (27–45 kg) dogs, using inulin and xanthan as comparators. Fecal samples from six dogs of each size group were evaluated. Overall microbiome composition, assessed using metagenomic sequencing, was shown to be driven mostly by dog size and not treatment. There was a clear segregation in the metabolic profile of the gut microbiota of small dogs versus medium-sized and large dogs. The fermentation of cRG-I specifically increased the levels of acetate/propionate-producing *Phocaeicola vulgatus*. cRG-I and inulin were fermented by all donors, while xanthan fermentation was donor-dependent. cRG-I and inulin increased acetate and propionate levels. The responses of the gut microbiota of different sized dogs to cRG-I were generally consistent across donors, and interindividual differences were reduced. This, together with the significant increase in *P. vulgatus* during fermentation in both this study and an earlier human ex vivo study, suggests that this abundant and prevalent commensal species has a core capacity to selectively utilize cRG-I.

## 1. Introduction

Gut health is the basis for general well-being and is determined by physiology, immune system activity, the resident gut microbiome, and dietary intake. Dietary fiber is a key factor that can alter the composition of the gut microbiome and modulate its metabolic function [1,2]. A balanced gut microbiome promotes host well-being by strengthening the intestinal barrier, participating in immune system modulation, providing protection from pathogens, and producing beneficial metabolites including short-chain fatty acids (SCFAs, mainly acetate, propionate, and butyrate) [3]. In dogs, gut microbiome imbalance, or dysbiosis, is associated with gastrointestinal disease (e.g., acute and chronic diarrhea, inflammatory bowel disease) [3], chronic kidney disease [4], abnormal behavior [5], anxiety behaviors [6], and metabolic and autoimmune disorders [7]. The main approaches to managing dysbiosis in veterinary medicine include dietary modulation with prebiotics/fibers or probiotics, treatment with antibiotics, or fecal microbiome transplantation [3]. Here, we focus on the ability of prebiotic dietary fibers to modulate the metabolic activity and community composition of the gut microbiota of small, medium-sized, and large healthy dogs.

The ability of colonic bacteria to utilize dietary polysaccharides depends on the presence of specific carbohydrate-active enzymes that digest certain linkage type(s) present in the fiber [8,9,10]. Simple carbohydrate structures with one monosaccharide type and one or two linkage types between sugars (e.g., fructooligosaccharides [FOSs], inulin, β-glucan from cereals) can be easily degraded by a wide variety of colonic bacteria, so the competition for these fibers is high, and their selectivity is low [11,12]. In contrast, complex fiber structures that contain numerous different monosaccharide types and many more linkage types (e.g., rhamnogalacturonan-I [RG-I], pectin, arabinoxylan, galactomannan, xyloglucan, xanthan) have higher selectivity because their degradation requires either large polysaccharide utilization loci that are found in, e.g., species of the genera *Bacteroides* and *Phocaeicola* [9,13] or intricate interactions between different bacterial species [11,12].

Studies with dietary fiber, aimed at modulating the gut microbiome composition and function, have shown great interindividual variation in response to some fibers, which could be due to baseline variation in the gut microbiome composition of the host and/or the selectivity of the dietary fiber being studied [8,11,12,14]. This has led to interest in strategically utilizing dietary fibers to reduce interindividual variability to stimulate more consistent outcomes and to investigate the possibility of selectively stimulating specific groups of colonic bacteria associated with health and well-being.

Carrot RG-I (cRG-I) is a complex and polydisperse pectin-derived polysaccharide fiber obtained by the enzymatic treatment of carrot pomace [15]. The structure of cRG-I is composed of four main monosaccharides, arabinose, rhamnose, galactose, and galacturonic acid, and over 17 distinct saccharide linkages [16]. Using in vitro models of the human intestinal tract, cRG-I has demonstrated prebiotic properties at a very low dose, with an increase in SCFA production over time, the stimulation of health-associated bacterial species, and improvement in the gut epithelial barrier integrity [17,18,19]. In contrast to inulin and xanthan, cRG-I was shown to reduce interindividual gut microbiota variability ex vivo [20], leading to more consistent results. Moreover, in a dietary intervention study in humans, low-dose cRG-I also had consistent modulatory effects on the gut microbiota [21] and beneficial immunomodulatory effects [22,23].

When developing prebiotic supplements for dogs, identifying fibers with an optimum selectivity may be advantageous to maintain universal applicability across different dog breeds, ages, and sizes. The gut microbiota of dogs can vary based on several characteristics, including size, breed, age, and body condition. One study of 96 dogs of different breeds, ages, and body condition scores suggests that age is more important in driving the gut microbiota community compared to breed or body condition score [24,25]. Another study with 83 dogs of different breeds and sizes found that there were differences in plasma metabolites and gut microbiome composition in small versus large dogs, potentially reflecting differences in their overall metabolism [24]. Considering a gene overlap of 63% between the dog and human microbiome, and that the diet can have a similar effect on the microbiota of dogs and humans [26], it is of interest to also evaluate the selective effects of different fibers on the gut microbiome of dogs [20].

After observing consistent effects across multiple enterotypes in humans, we were interested in investigating the effects of low-dose cRG-I on the gut microbiome of dogs and learning how the results in dogs of different sizes would compare. Specifically, we wanted to evaluate whether low-dose cRG-I modulates the composition and metabolic function of the dog gut microbiome and how the fermentation of cRG-I compares to that of inulin at the same low dose. We also wanted to understand whether the effect of low-dose cRG-I was also consistent across dog sizes and whether there is a responder/non-responder effect of xanthan in dogs, as observed in humans. Therefore, the aim of this study was to investigate the effects of low-dose cRG-I on the microbial composition and metabolite production of the gut microbiota of small, medium-sized, and large dogs using short-term batch fermentation technology [27]. In addition, the selectivity of cRG-I was compared with inulin and xanthan as examples of low- and very-high-selectivity fibers, respectively.

## 2. Materials and Methods

### 2.1. Canine Donors

Fecal samples were collected from six small dogs (5–10 kg), six medium-sized dogs (10–27 kg), and six large dogs (27–45 kg). All dogs were in good physical health at the time of donation; the dogs had no acute or chronic clinical signs or physical conditions that they were currently under treatment for. Pet owners were instructed to collect a small amount of their dog’s fecal sample and place it in a container equipped with an Oxoid^TM^ AnaeroGen^TM^ sachet (Thermo Fisher Scientific, Waltham, MA, USA). Fecal samples were shipped to the ProDigest facilities within 24 h of collection and stored at 4 °C upon arrival. All samples were processed within 48 h post-defecation. Pet owners provided information about their dog’s breed, age, body weight, gender, health status, and diet and supplement use. Nine different breeds represented small, medium-sized, and large dogs (Pomeranian, Chihuahua, Jack Russel, West Highland White Terrier, Beagle, Golden retriever, Belgian shepherd, Saint Bernard, rescue/unknown). The dogs were aged between 1 and 11 years (median 6 years) and were a mix of male (67%) and female (33%), and the majority (83%) consumed kibble-based diets (at least five different kibble formulations/brands), while the rest of the dogs consumed ‘wet’ dog food, and none of the dogs consumed a ‘raw’ diet only. Fecal samples were prepared under anaerobic conditions and mixed with a modified version of the cryoprotectant developed by Hoefman et al. [28] (the cryoprotectant was sparged by bubbling nitrogen gas through the liquid for 30 min to displace oxygen and establish an anaerobic environment). Next, the samples were aliquoted, flash-frozen, and stored for a maximum of 5 months at −80 °C. To preserve the function of the intestinal bacteria, samples were defrosted immediately prior to use.

### 2.2. Test Compounds

**Figure 1:**
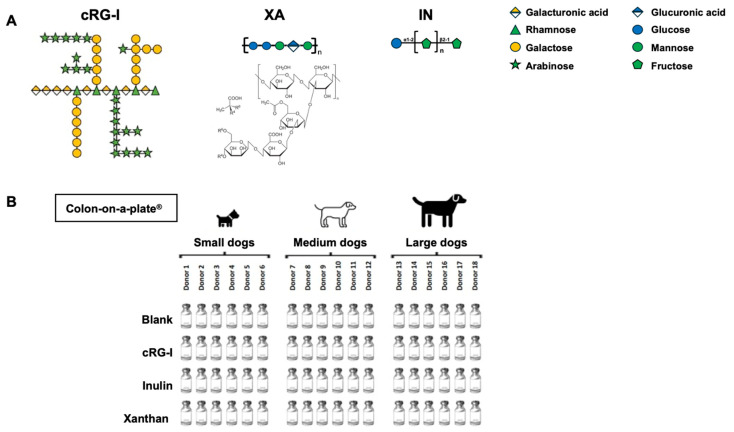
Elements of study design. (**A**) Chemical structures of test fibers. (**B**) Experimental setup. cRG-I, carrot rhamnogalacturonan-I; IN, inulin; XA, xanthan.

This study evaluated the same three test compounds that were used in an earlier ex vivo study with human fecal samples [20]: cRG-I, which is a natural extract from *Daucus carota* subsp. *sativus* (Benicaros^®^; NutriLeads, Wageningen, The Netherlands); inulin from chicory (I2255, Merck, Overijse, Belgium); and xanthan (3557, Carl Roth, Karlsruhe, Germany), all of which were supplied as powder. cRG-I is a water-soluble non-digestible fermentable fiber upcycled from carrot pomace and highly enriched (80%) in the cRG-I domain of pectin. The extraction method and characteristics of cRG-I were described earlier [15]. The monosaccharide composition of cRG-I is as follows (% mol/mol): rhamnose, 14.3; arabinose, 34.8; galactose, 19.6; fucose, 0.8; glucose, 4.3; mannose, 0.9; xylose, 0.7; galacturonic acid, 25. Inulin is a polymer of (2,1)-bond-linked fructose residues with a chain-terminating glucose with a fructose–glucose ratio of 20:1. Xanthan consists of repeated pentasaccharides of (1,4) glucose moieties decorated with trisaccharide side chains comprising 2 mannose and 1 glucuronic acid unit. The chemical structure of each test product fiber is shown in Figure 1A.

### 2.3. Short-Term In Vitro Batch Fermentations

At the start of the experiment, mini-reactors with 15 mL reactor volumes were filled with a fiber-depleted nutritional medium buffered at pH 6.50 ± 0.20 (PD001; ProDigest, Gent, Belgium). Prior to the experiment, this nutritional medium was made anaerobic by boiling to drive out oxygen. Next, individual test products were added to single reactors at a final concentration of 1.5 g/L. Blank wells did not receive a test product (i.e., no substrate control). Cryopreserved fecal suspensions (7.5% *w*/*v* fecal material) were added to each well (10% *v*/*v*), serving as the microbial source. Plates were incubated in an anaerobic atmosphere at 39 °C for 48 h. The experimental setup is shown in Figure 1B. Samples were collected 48 h after the start of the experiment and assessed for pH; community composition; the levels of SCFAs (acetate, propionate, and butyrate), lactate, BCFAs, and ammonium; and metabolic fingerprint using laser-assisted Rapid Evaporative Ionization Mass Spectrometry (LA-REIMS).

### 2.4. Microbiota Composition Analysis

DNA was extracted from microbial pellets generated during ex vivo fermentations using a PowerFecal DNA extraction kit under the standard instructions from the manufacturer (Qiagen, Germantown, Maryland, MD, USA).

DNA sequencing was performed using Oxford Nanopore Technologies equipment and consumables (Oxford Nanopore Technologies, Oxford, UK). Post-extraction DNA was prepared using the rapid barcoding 96 kit version 14 (SQK-RBK114.96). The final pool of barcoded samples was added to a GridION flow cell (R10.4.1). GridION reads were base-called using the Super Accuracy Analysis protocol, achieving an estimated accuracy of 99.5%. Adaptor sequences were subsequently trimmed using Porechop v0.2.4 [29]. Quality filtering was performed with Chopper v0.5.0, retaining reads with a minimum length of 300 base pairs and a sufficient PHRED score [30]. These high-quality (“HQ”) reads were used for downstream analysis.

For microbiome profiling, the taxonomic classification of metagenomic reads was performed using Kraken2 v2.0.7, referencing a database made from dog-derived bacterial genomes downloaded from PATRIC databases (downloaded 19 March 2024) [31]. Subsequently, Bracken v2.5.0 was employed to estimate relative abundance profiles at various taxonomic levels, providing a semi-quantitative assessment of microbial community composition [32]. These derived microbiome profiles served as the foundation for subsequent analyses. Alpha diversity (species richness) was calculated via the Shannon diversity index which captures both richness and evenness, providing a comprehensive view of microbial community structure. This metric is linked to microbiome function and stability, offering insights into health outcomes. As an established ecological index, it facilitates standardized comparisons across studies [33]. Further, beta diversity (dissimilarity between samples) was assessed using the Bray–Curtis index [34].

### 2.5. Microbial Fermentation/Metabolic Activity Analysis

pH was measured using a Rapid pH™ automated benchtop pH meter (Hudson Robotics, Springfield, NJ, USA). Gas pressure was measured using a hand-held pressure indicator (CPH6200; WIKA, Lawrenceville, GA, USA) with a transmitter (CPT6200; WIKA). Acetate, propionate, butyrate, and branched-chain fatty acids (BCFAs, isobutyrate, isovalerate, and isocaproate) were assessed using capillary gas chromatography coupled with a flame ionization detector as described by De Weirdt et al. [35]. Lactate was measured using the Enzytec^™^ assay kit (R-Biopharm, Darmstadt, Germany). Ammonium levels were assessed using the indophenol blue spectrophotometric method as described by Tzollas et al. [36]. For each assay, measurements were performed in a single repetition.

### 2.6. Metabolic Fingerprinting with LA-REIMS

Samples were thawed at 4 °C and briefly vortexed (400 rpm, 20 °C, 1 min). A volume of 100 µL was transferred into a well of a 96-well plate and subjected to LA-REIMS analysis. The LA-REIMS platform used a MID infrared laser system (OpoletteTM HE2940; OPOTEK, LLC, Carlsbad, CA, USA) that consisted of a Q-switched Nd:YAG laser pumping Optical Parametric Oscillator (OPO). The transmission of the laser energy (wavelength of 2.94 µm, 175 µs pulse time) into the sample was achieved through free space optics, including a series of metallic-coated mirrors (OptoSigma Global Top, Les Ulis, France) and a Plano-convex lens (Thorlabs, GmbH, Bergkirchen, Germany). Mass analysis was carried out by means of a Xevo G2-XS Quadrupole Time-of-Flight (QToF) mass spectrometer (Waters Corporation, Wilmslow, UK), being operated in negative ionization mode and applying an *m*/*z* scan range from 50 to 1200 Da. The main parameters concerned a cone and heater bias voltage of 50 V, a scan time of 0.3 s, and an isopropanol flow of 0.25 mL min^−1^. More detailed information on the LA-REIMS analysis as well as associated data processing can be retrieved from Plekhova et al. [37].

### 2.7. Statistical Analysis

All statistical analyses and visualizations for the metagenomics data were performed using R (version 4.3.2) and the following packages: stringr, vegan, ape, dplyr, tidyr, ggplot2, mclust, pheatmap, ggpubr, Rtsne, scales, xgboost, and caret.

To analyze the microbiota composition data, the species-level count table was filtered to remove extraction and sequencing controls. Amplicon sequence variants (ASVs) with zero reads after control removal were also eliminated. Samples with a total read count below 1000 were excluded from further analysis. The remaining ASV counts were normalized using a centered log-ratio (CLR) transformation to account for compositional data properties. Bray–Curtis dissimilarity was calculated with Principal Coordinate Analysis (PCoA) using the vegan package on rarefied ASV count data, with rarefaction performed to the smallest read count across samples. PCoA was then performed using the ape package to visualize beta diversity patterns. The significance of PCoA axes in differentiating treatment groups was assessed using Mann–Whitney U tests, implemented via the run_mannwhitney function and visualized using the plot_top_axis function. Bray–Curtis distances within each treatment group were calculated to assess the homogeneity of microbial communities within treatments (beta diversity). Statistical comparisons between treatment groups were performed using Wilcoxon signed-rank tests. Shannon diversity indices were calculated using the vegan package (alpha diversity). The relative abundances of prevalent species (present in at least 8 samples) and genera were calculated. Kruskal–Wallis tests were used to identify species and genera with significant differences in relative abundance across treatment groups. Heatmaps were generated using the pheatmap package to visualize the relative abundances of the top 50 species and top 25 genera. An XGBoost decision tree model was trained using the caret package to identify species and families that best differentiate between the cRG-I and inulin treatment groups. The importance of each feature (species or family) was assessed using the xgboost package.

Differences in fermentation/metabolic activity between the test product and blank were assessed using paired two-sided Student’s *t*-tests. For the analysis, per-donor measurements were considered as replicate values, resulting in six replicate measurements per donor category (small, medium-sized, or large dogs). This approach accounts for individual differences by only considering an effect significant if it is observed over multiple donors. A *p*-value of <0.05 was considered statistically significant.

For the analysis of the LA-REIMS data, normalized data were subjected to multivariate statistical analysis using SIMCA 17 (Sartorius, Göttingen, Germany). Data were pre-processed, thereby performing log transformation to induce normal distributions and unit variance scaling (1/standard deviation [SD]) to standardize the range of signal intensities. Unsupervised Principal Component Analysis (PCA-X) was executed to assess the natural patterning of samples and reveal potential outliers (based on Hoteling’s T2 criterion). Orthogonal Partial Least Squares Discriminant Analysis (OPLS-DA) was used to differentiate samples according to the experimental conditions in a supervised fashion. The validity of the OPLS-DA models was verified by permutation testing (*n* = 100), a cross-validated analysis of variance (*p*-value < 0.05), and the quality parameter Q2(Y) (≥0.5). In general, the model performance is described by R2(X) (the predictive and orthogonal variation in X-values, i.e., *m*/*z* features), R2(Y) (the ability to predict the Y-data for the specifically used dataset, i.e., predicting the sample classification), and Q2 (the ability to correctly predict the Y-data when an external dataset would be considered).

## 3. Results

### 3.1. Effects on Microbial Diversity and Composition

**Figure 2:**
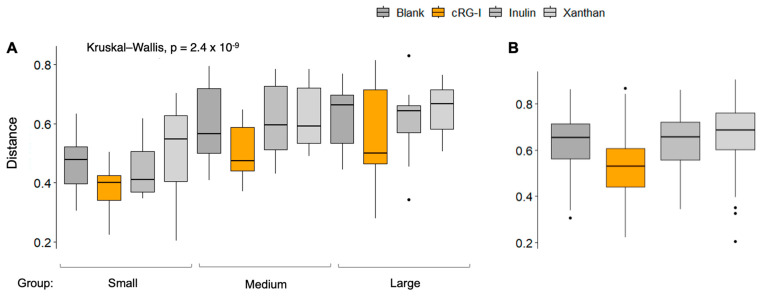
Changes in microbial community diversity. (**A**) Alpha diversity (Shannon index). (**B**) Beta diversity (Bray–Curtis index) within cRG-I treatment group was significantly smaller than that within other conditions (*t*-test, *p*-values < 0.01). Black dots in the figure represent outliers. cRG-I, carrot rhamnogalacturonan-I.

The alpha diversity, a measure for species richness, was maintained for all the test products compared to the blank (Figure 2A). The beta diversity, a measure for the dissimilarity between donors within a treatment group, was significantly decreased in the cRG-I group compared to the blank (Figure 2B), suggesting that cRG-I has a stabilizing effect on the gut microbiota of dogs by lowering the interindividual differences. The beta diversity index for inulin and xanthan remained unchanged compared to the blank.

**Figure 3:**
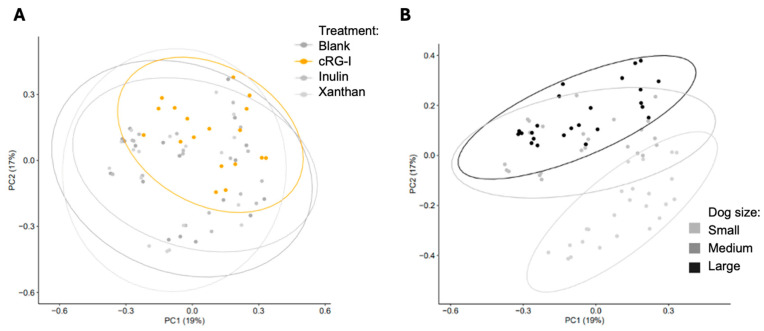
Changes in microbial community composition. (**A**) PCA plots by test article. (**B**) PCA plots by dog size. cRG-I, carrot rhamnogalacturonan-I.

Treatment effects on microbial composition were assessed next. Initial PCA plots demonstrate an overlap in the microbial composition according to treatment (Figure 3A), but interestingly there is a clustering according to dog size (Figure 3B), suggesting that the overall microbiome composition is driven by dog size rather than by the treatments.

**Figure 4:**
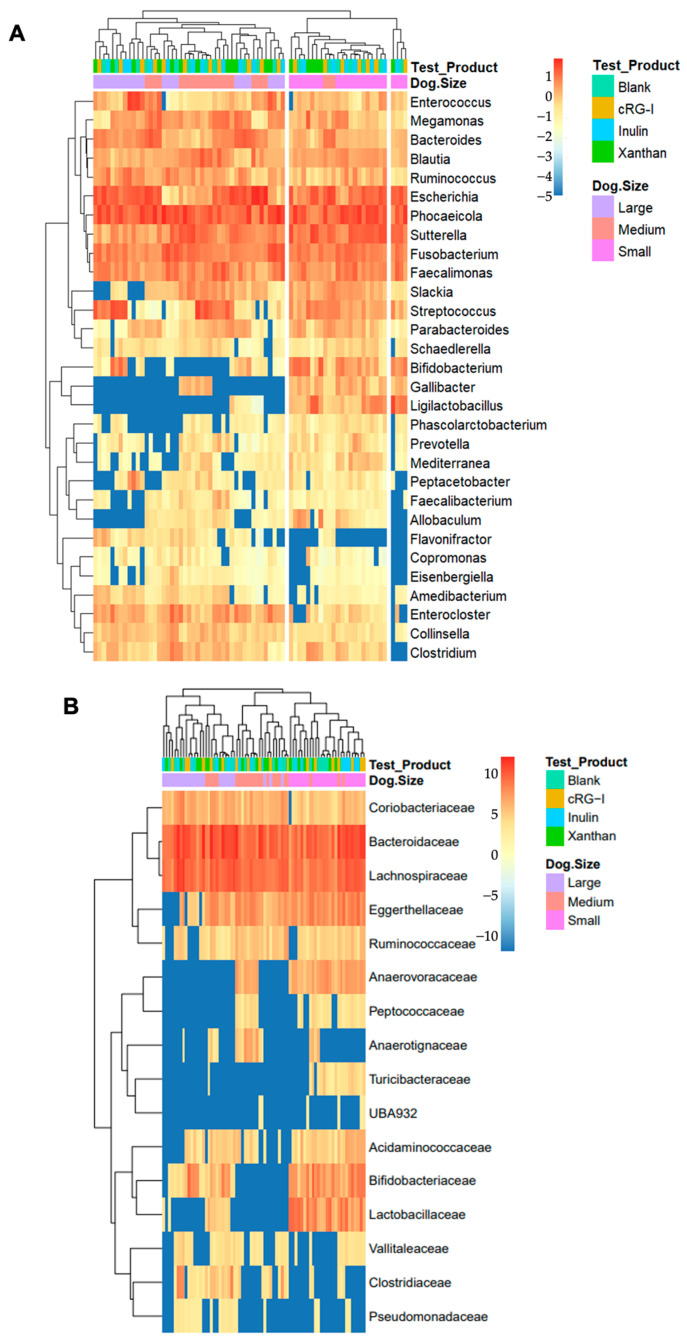
Heatmaps depicting microbial abundance. (**A**) The relative abundance of the top 25 most abundant genera. (**B**) The relative abundance of key families. Kruskal–Wallis, *p* < 0.05, size_treatment (e.g., cRG-I_small vs. cRG-I_large). cRG-I, carrot rhamnogalacturonan-I.

This was further visualized with a heatmap of the log10 relative abundance (with the addition of a 0.00001 pseudocount) of the top 30 bacterial genera (Figure 4A) and bacterial families (Figure 4B). Interestingly, in this cohort, the medium-sized and large dogs seem to lack *Ligilactobacillus* (formerly *Lactobacillus) animalis* and *Bifidobacterium* spp., and the sequencing data demonstrates a clustering of the small dog group while using unsupervised methods.

**Figure 5:**
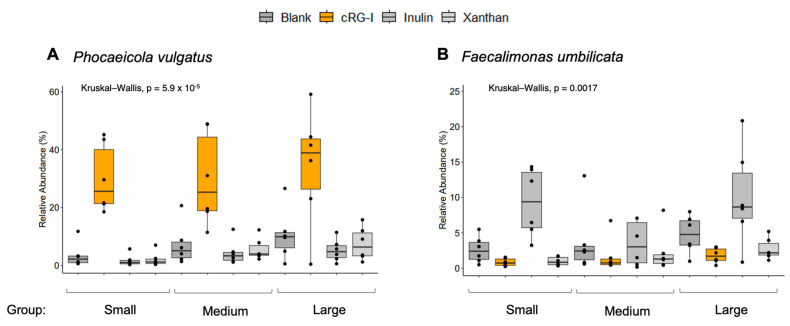
Relative abundance of (**A**) *Phocaeicola vulgatus* per treatment group and (**B**) *Faecalimonas umbilicata* per treatment group. Black dots in the figure represent outliers. cRG-I, carrot rhamnogalacturonan-I.

When looking at the whole cohort, *Phocaeicola vulgatus* (formerly known as *Bacteroides vulgatus*), *Faecalimonas umbilicata*, *Collinsella intestinalis*, *Phocaeicola plebeius*, and *Ruminococcus B gnavus* were the top five most differentiating species (Supplementary Figure S1). From the top three most differentiating species, Figure 5A shows how cRG-I specifically increased the relative abundance of *P. vulgatus* in all dog size groups, with consistent effects (i.e., 6/6 donors) in the medium-sized and small dogs and in 5/6 large dog donors. Figure 5B shows that inulin selectively increased the abundance of *F. umbilicata* across the groups. *C. intestinalis* was not selectively increased by treatment but did appear to have a lower abundance in smaller dogs (Supplementary Figure S2).

Many of the species with a >1.5-fold increase in relative abundance compared with blank were unique for each of the three supplements (cRG-I, eight unique species; inulin, six unique species; xanthan, nine species) (Table 1). With all three supplements, the number of increased species was the highest for the small dog group compared with the medium-sized and large dog groups.

### 3.2. Effects on Microbial Fermentation/Metabolic Activity

Monitoring the pH during a colonic incubation provides an indication of the production of microbial metabolites like SCFAs, lactate, and ammonium. The primary objective of pH measurement is to ensure the maintenance of physiological pH conditions throughout the incubation period. During the colonic incubations, the reduction in pH values was similar for cRG-I and inulin compared with the blank after 24 h and 48 h, demonstrating that each of the test products was fermented by the microbiota of dogs in each of the size categories (Table 2). No change in pH was observed in the medium-sized dogs treated with xanthan, with only 1/6 dogs having a response. A very small decrease in pH was observed in the small and large dogs. The starting pH in each Colon-on-a-plate^®^ well was 6.50 ± 0.20, and the lowest pH measured at the end of the experiment was 6.04, which was well within the in vivo pH range (pH 5.0–6.9) [38], indicating that pH levels remained optimal throughout the experiment.

Like pH, gas production is a measure of overall microbial activity. Gases are produced during saccharolytic and proteolytic fermentation. Hence, the process can be considered a marker of overall microbial activity (saccharolytic and proteolytic), being inherent to gut microbial fermentation activity. Overall, the impact of the test products on gas production was comparable across different dog sizes. cRG-I and inulin stimulated gas production across all dog sizes, indicative of enhanced microbial activity, with a significant increase at 48 h (small dogs, 64% and 68%, respectively; medium-sized dogs, 63% and 60%; large dogs, 54% and 61%). However, xanthan had only a mild impact on gas production, with a significant increase of 18% and 9% with the microbiota of small and large dogs, respectively, and no significant change with the microbiota of medium-sized dogs (2/6 dogs had a response [>5% increase from baseline]), suggesting a mild impact on microbial activity in the responders.

**Figure 6:**
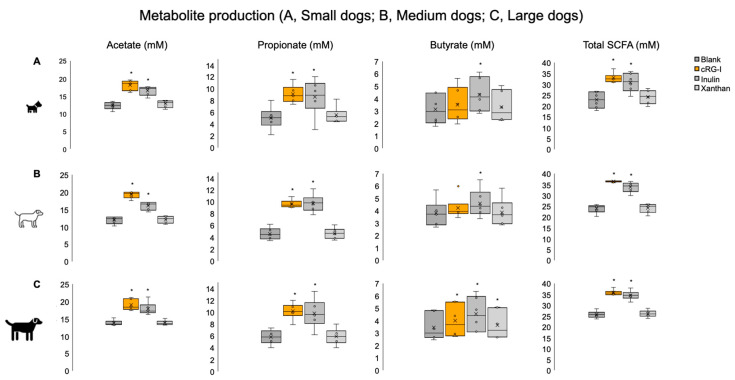
SCFAs acetate, propionate, and butyrate and total SCFA levels at 48 h in small (**top row**), medium-sized (**middle row**), and large (**bottom row**) dogs. * *p*-value < 0.05 versus blank (paired two-sided Student’s *t*-test). Orange dot in the figure represents an outlier. cRG-I, carrot rhamnogalacturonan-I; SCFA, short-chain fatty acid.

In the small dog population, cRG-I and inulin supplementation resulted in a significantly greater production of acetate and propionate compared with the blank at 48 h (Figure 6, top row). With cRG-I supplementation, there was a 47% increase in acetate and a 79% increase in propionate versus the blank. With inulin, the increase versus the blank was 34% for acetate and 70% for propionate. Changes in butyrate levels were highly donor-dependent, and a significant increase in production versus the blank was only achieved with inulin supplementation. The findings were similar for medium-sized dogs, with an increase in acetate versus the blank of 61% for cRG-I and 34% for inulin and respective increases in propionate of 100% and 112% (Figure 6, middle row). Across these donors, butyrate production was significantly increased versus the blank with inulin (21% increase). cRG-I supplementation resulted in donor-dependent increases in butyrate but did not reach significance across donors. Among large dogs, cRG-I and inulin similarly increased acetate and propionate production versus the blank (Figure 6, bottom row). Butyrate production was significantly increased versus the blank for all test products (cRG-I, 15%; inulin, 31%; xanthan, 5%). The results for total SCFA production showed significant increases in cRG-I and inulin for all dog sizes (Figure 6, right-hand column). Xanthan supplementation had no/little effect on the production of SCFAs in any of the groups, with only 1/6 dogs in the small dog group responding to xanthan (0/6 dogs in the medium-sized or large dog groups had a response). Data for individual donors is shown in Supplementary Figure S3.

After 48 h, lactate was largely consumed by most donors in the small and large dog groups but was not fully consumed by most donors in the medium-sized dog group. No significant differences in lactate levels between any of the test treatments and blank were observed.

BCFAs and ammonium are markers of protein metabolism. Donor-dependent differences were observed for both BCFA and ammonium production at 48 h. No significant differences between BCFA production with any of the test products versus the blank were observed in the small or large dog group (Supplementary Figure S4). However, in the medium-sized dogs, BCFA production was significantly reduced versus the blank with inulin supplementation (6/6 dogs), and BCFA production was also reduced in the cRG-I group (5/6 dogs) but did not reach significance. No significant differences between ammonium production with any of the test products versus the blank were observed in the small dog group. In samples from the medium-sized dogs, ammonium production was significantly decreased versus the blank with cRG-I supplementation (6/6 dogs) and reduced in the inulin group without reaching significance (5/6 dogs). Interestingly, ammonium was significantly increased versus the blank with xanthan supplementation. In large dogs, ammonium production was significantly decreased versus the blank with cRG-I and inulin supplementation and significantly increased with xanthan.

### 3.3. Effects on Metabolic Fingerprint (LA-REIMS)

**Figure 7:**
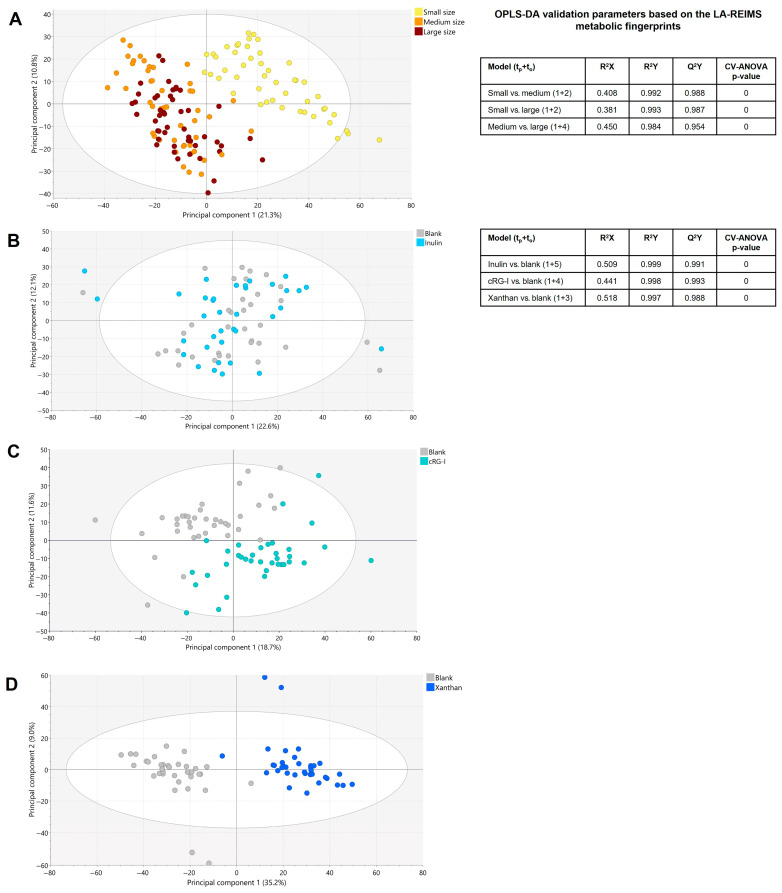
PCA-X-score plot based on LA-REIMS data (24 h and 48 h). (**A**) All biological samples (*n* = 144). (**B**) Blank versus inulin. (**C**) Blank versus cRG-I. (**D**) Blank versus xanthan. Associated iQC-samples were excluded from analysis. LA-REIMS metabolomic data were generated in negative ionization mode. OPLS-DA validation parameters for evaluation of various dog sizes and different treatments based on LA-REIMS metabolic fingerprints are also shown. ANCOVA, analysis of covariance; cRG-I, carrot rhamnogalacturonan-I; iQC, internal quality control; LA-REIMS, laser-assisted rapid evaporative ionization mass spectrometry; OPLS-DA, orthogonal partial least squares discriminant analysis; PCA-X, unsupervised principal component analysis, t time; Q^2^Y quality parameter; R^2^X the model performance; R^2^Y the ability to predict the Y-data for the specifically used dataset.

Metabolic fingerprinting via LA-REIMS analysis targets a broad metabolic spectrum beyond changes in typical fermentation parameters and can provide comprehensive insights into the interactions between test products and gut microbes. An LA-REIMS analysis of all biological samples (24 h and 48 h) showed a clear segregation of the metabolic profile of the gut microbiome of small dogs compared with that of medium-sized and large dogs (Figure 7A). Metabolic differences between the gut microbiome of medium-sized and large dogs were also demonstrated. Supervised OPLS-DA modeling confirmed that these metabolic differences between the small dogs and medium-sized/large dogs, as well as between the medium-sized and large dogs, were significant. When comparing treatments to the blank, the metabolic fingerprint showed increased segregation with increasing fiber selectivity/complexity (i.e., inulin to cRG-I to xanthan) (Figure 7B–D). Significant metabolic differences between 24 h and 48 h were observed in all groups, indicating a time effect. Although metabolic alterations were revealed during the course of the experiment for all experimental conditions under evaluation (i.e., blank, inulin, cRG-I, and xanthan), the treatment effects as manifested for the different treatments compared to the blank were similar at the 24 h and 48 h time-points.

## 4. Discussion

Diet influences the composition, diversity, and metabolic capacity of the gut microbiota. Thus, there is great interest in developing targeted dietary interventions that can modulate the gut microbiota to improve health and reduce disease risk. The functions of the gut microbiota are similar in humans and dogs, and they include immunomodulation, contributions to host metabolism, and protection against pathogens [39]. Other gut microbiota similarities between humans and dogs include an overlap in gene content [26], similar indicators of dysbiosis (e.g., an increased abundance of Enterobacteriaceae [40,41]), responsiveness to nutrients [42], and a similar response to dietary changes [26]. Changes in the diet, such as an increase or decrease in the consumption of dietary fiber, can have a significant impact on both the composition and metabolic activity of the gut microbiota [43], which can only be maintained with long-term dietary adaptation [42]. Considering that the diet of dogs is far less varied than the diet of humans, it is important to ensure their diet contains components to support a healthy gut microbiome. The gut microbiota of healthy dogs comprises three main phyla, Firmicutes, Bacteroidetes, and Fusobacteriota, which include many SCFA-producing species [44,45]. Several diseases are associated with gut microbiome dysbiosis, highlighting the importance of dietary adaptations to promote gut microbiome balance. For example, dogs with gastrointestinal disease have reduced microbiome diversity; a reduction in some core species, including *Fusobacterium* spp. and *Clostridium hiranonis*; and subsequently a reduction in SCFA levels [39,46,47,48]. Of note, dietary-induced changes to the gut microbiome are reported to be independent of dog breed [26], but less is known about the influence of dog size.

cRG-I is a dietary fiber that has demonstrated beneficial effects on the composition and function of the gut microbiota in both preclinical and clinical studies, via the selective stimulation of taxa that are reported to be consistently present in healthy human adults [19,20,21]. After observing a consistent effect of low-dose cRG-I in humans across multiple enterotypes, we were interested in investigating the effects of low-dose cRG-I on the gut microbiome of dogs. Previous studies conducted in healthy dogs, dogs with food responsive enteropathy, and dogs with mild inflammatory bowel disease have demonstrated that supplementation with a range of prebiotics, including mannooligosaccharides (MOSs), galactooligosaccharide (GOSs), β-glucans, and prebiotic blends (FOS + MOS + GOS + β-glucan; β-glucan + MOS), did not result in significant differences in fecal SCFA production (acetate, propionate, butyrate) in dogs who received prebiotics compared with placebo [49,50]. However, one study investigating the effects of daily GOS supplementation in healthy dogs reported a significant increase in fecal acetate and propionate concentrations compared with baseline levels [51]. Our in vitro study demonstrated a significant increase in acetate and propionate production with cRG-I supplementation versus the blank in all dog sizes. A significant increase in butyrate was also observed in the large dog group. The production of these SCFAs suggest potential beneficial effects on health with cRG-I supplementation, and this needs to be validated in future feeding studies. Similarly, there was a significant increase in these SCFAs with inulin compared with the blank for all dog size groups. In comparison, xanthan had little effect on SCFA production. The separation in metabolite production between supplements and control, observed with LA-REIMS in this study, was likely due to the increasing fiber selectivity/structural complexity (INS > cRG-I > XA) also reported earlier [20]. Regarding species diversity, β-glucan supplementation in dogs with mild inflammatory bowel disease resulted in an increase in the Shannon alpha diversity index [49]. This contrasts with the present in vitro study, where prebiotic supplementation did not impact alpha diversity. The different SCFA and Shannon diversity results may be explained by differences in the digestibility of the various test fibers, experimental differences (in vivo versus in vitro study designs), the duration of fiber supplementation, or differences in donor health status.

This study demonstrated that overall, dog size was the greatest predictor of functional response. In particular, the small dog population had a distinct microbial community composition compared with the medium-sized and large dog populations, e.g., smaller dogs had a lower abundance of *Collinsella intestinalis* than larger dogs, also observed by other researchers [24]. Interestingly, for all three supplements, the number of increased species was the highest for the small dog group compared with the medium-sized and large dog groups. LA-REIMS analysis revealed a distinct metabolic response for small dogs compared with medium-sized and large dogs. The OPLS-DA modeling of the evaluation of LA-REIMS data by treatment indicated that the interpretation of these results should be made with care, as there was limited statistical importance given to the design of the multilevel dataset for direct pairwise comparisons. However, combined with the metagenomic data, these observations do highlight the diversity in metabolic and community composition response to fibers according to dog size. As noted previously, it has been reported that there are differences in the fecal microbiome composition between small and large dogs [24]. Further, a study in healthy dogs showed individualized responses to a combination prebiotic/probiotic supplement and reported that the magnitude of response (beta diversity) was associated with the baseline gut microbiome composition [52]. This suggests that differences in the composition of the baseline gut microbiome may, at least in part, explain the observation that size was the greatest predictor of response. As with any study conducted using in vitro models, the findings are not directly applicable to the in vivo situation, and this study had a small number of dogs per group (*n* = 6), which may limit the generalizability of the results. Dedicated intervention studies are needed to better understand the potential beneficial effects of cRG-I in dogs.

A cRG-I dose of 1.5 g/L was chosen based on previous studies. These studies included one ex vivo study that had a similar design to our study but used the gut microbiota of humans [15] and one dietary intervention study in humans. The intervention study showed that daily doses of 0.3 g/day or 1.5 g/day cRG-I effectively modulated gut microbiota composition and immune responsiveness to rhinovirus [15,22,23]. In the ex vivo study utilizing human fecal samples, cRG-I supplementation had a homogenizing effect on the gut microbiota composition, with a significant decrease in beta diversity compared with the blank control [20]. The level of interindividual variability in SCFA production following cRG-I supplementation was also low, further suggesting a consistent effect of fiber across enterotypes. The present study demonstrated similar results with the dog gut microbiota, reporting low interindividual variability in response to cRG-I supplementation with a significant reduction in beta diversity compared with the blank for the SCFA data. Inulin, a common prebiotic fiber with low selectivity was fermented by all donors in both the human study and the present dog study; however, in both studies there was a high level of interindividual variability in the response [20]. In contrast, xanthan is a fiber with high selectivity and was not fermented by all donors in either study, demonstrating inconsistency in outcomes with a mix of responders and non-responders for both humans and dogs [20]. The variability in response to inulin and xanthan highlights the novelty of the cRG-I fiber that induces responses with minimal interindividual variability. Such a prebiotic fiber with a consistent and specific response across a broad target population could allow manufacturers to focus on the application of one ingredient across a wide range of products and give confidence to pet parents that the product will be effective for their pets.

*P. vulgatus* was the most differentiating bacterial species in this canine cohort and was consistently and selectively increased by cRG-I in all dog size groups, suggesting that it is likely a keystone species for cRG-I fermentation in dogs [53]. Interestingly, this is also one of the key species that was consistently increased in the human ex vivo study [20] and was correlated to outcomes in the intervention study in humans [21]. *P. vulgatus* is a keystone species that possesses a broad range of carbohydrate-active enzymes (CAZymes) within its polysaccharide utilization loci (PUL) [13], potentially playing an important role in degrading cRG-I into smaller parts, enabling cross-feeding within the gut metabolic network. *Phocaeicola* spp. have been associated with both health and disease depending on the strain, their intestinal location, and host health status [54,55]. For example, *P. vulgatus* is known to produce acetate, propionate, butyrate, succinate, and valeric acid [56,57]. These SCFAs provide a source of energy for colonocytes and have anti-inflammatory potential [58]. In mice, *P. vulgatus* is beneficial for maintaining intestinal barrier function and preventing atherosclerosis by reducing gut microbial lipopolysaccharide production [54,59]. It was also recently shown to attenuate the development of metabolic dysfunction-associated steatotic liver disease (MASLD) when applied as a probiotic in a mouse model of MASLD [60]. However, an increased abundance of *P. vulgatus* was observed in the feces of patients with type 2 diabetes [61]. Additionally, reduced levels of *Phocaeicola* spp. have been reported in dogs with chronic enteropathies and in Yorkshire terriers with inflammatory bowel disease [62,63]. Other species that had an increased relative abundance with cRG-I supplementation included *Paraprevotella*, *Bifidobacterium pseudolongum*, *Blautia*, *Fournierella*, and *Phascolarctobacterium*. In humans, *Paraprevotella* has an immunomodulatory effect by protecting IgA from trypsin degradation [64] and may have a protective effect on cognitive performance [65] and against some cancers [66]. However, an increased abundance has been reported in people with depression [67] and children with autism spectrum disorder [68]. In mice, *B. pseudolongum* has demonstrated prebiotic properties, such as helping to restore a healthy gut microbiome, improving intestinal barrier function, and producing acetate [69]. *Blautia* spp. are involved in immunomodulation and produce SCFAs, which have important host health benefits [70]. *Fournierella* spp. have demonstrated an anti-inflammatory effect that is mediated by their production of indole-3-propionic acid in mice [71]. Finally, *Phascolarctobacterium* can convert succinate to propionate [72] and has been associated with a positive mood [73]. Future in vivo validation, by means of feeding studies, are needed to evaluate the full beneficial potential of cRG-I in pets.

Supplementation with a low-dose prebiotic like cRG-I may be attractive to dog owners, as it is a complex fiber that is initially degraded by *P. vulgatus*, a bacterial species that was present in all dog size groups. In this in vitro study, the effects of cRG-I were consistent across all dog size groups, and there was a reduced level of interindividual variation in response compared with inulin and xanthan. Further, previous studies in humans have demonstrated several health-related benefits with cRG-I supplementation [22,23]. These include the acceleration of the innate immune and antiviral responses to rhinovirus infection and a shortened duration and severity of common cold symptoms. Given the similarity in the gut microbiome and response to dietary changes in humans and dogs [26], the health benefits observed in humans may be similar in dogs.

## 5. Conclusions

In this study, it appears that dog size is a key driver of fecal microbiome and metabolome responsiveness to dietary fiber; however, dog size might simply be a proxy for a combination of breed/physiology (pH and gastric transit time), breed/genetics (immune system), diet (choice of diet based on size), and other factors. This study also showed that a low dose of cRG-I reduced interindividual differences between dogs in various size categories through consistent effects on gut microbiota composition and function. This effect of cRG-I on reducing interindividual variability suggests that dietary interventions with this prebiotic fiber may yield more consistent outcomes across donors by modulating the metabolic network in the same direction. The finding that *P. vulgatus* was significantly and consistently increased in all donors during the fermentation of cRG-I, in this and an earlier human ex vivo study, suggests that this abundant and prevalent commensal acts as a keystone species with a core capacity to selectively initiate the hydrolysis of cRG-I, thus releasing smaller fragments that can be fermented by other members of the gut microbiota.

## Figures and Tables

**Table 1:** Species with a >1.5-fold increase in relative abundance compared to the blank.

Species with Increased Relative Abundance Compared to Blank	cRG-I			Inulin			Xanthan		
	S	M	L	S	M	L	S	M	L
*Phocaeicola vulgatus*	↑	↑	↑						
*Schaedierella glycyrrhizinilytica A*		↑	↑						
*Paraprevotella* *cluster 73*	↑								
*Bacteroides fragilis A*	↑			↑					
*Bifidobacteria pseudolongum*	↑								
*Bifidobacterium globosum*	↑								
*Blautia A cluster 62*	↑							↑	
*Blautia A cluster 63*		↑	↑		↑				
*Blautia A cluster 64*		↑	↑						
*Blautia A sp900541345*	↑	↑		↑	↑				
*Fournierella sp002160145*	↑								
*Phascolarctobacterium A* *sp900552855*	↑			↑	↑				
*Schaedierella sp900765975*	↑	↑							
*Faecalimonas umbilicata*				↑	↑	↑			
*Ruminococcus B gnavus*				↑		↑			
*Schaedlerella cluster 57*				↑					
*Lactobacillus acidophilus*				↑					
*Phocaeicola sp900546645*				↑					
*Phocaeicola sp900544075*				↑					
*Clostridium Q sp000435655*								↑	↑
*Sutterella sp900754475*							↑		
*Ligilactobacillus animalis*							↑		
*Streptococcus dysgalactiae*							↑		
*Streptococcus pseudoporcinus*							↑		
*Catenibacterium sp000437715*							↑		
*Clostridium Q cluster 49*							↑		
*Enterocloster sp001517625*								↑	↑
*Pseudomonas aeruginosa*									↑

↑ indicates an increase; cRG-I, carrot rhamnogalacturonan-I; L, large; M, medium; S, small.

**Table 2:** Average pH and gas production over time.

	Small Dogs				Medium-Sized Dogs				Large Dogs			
	BL	cRG-I	IN	XA	BL	cRG-I	IN	XA	BL	cRG-I	IN	XA
pH												
24 h	6.49	6.33	6.29	6.46	6.53	6.34	6.37	6.48	6.54	6.34	6.33	6.47
48 h	6.49	6.31	6.30	6.46	6.24	6.07	6.09	6.25	6.30	6.10	6.11	6.24
Gas production (kPa)												
24 h	4.53	5.23	4.87	6.12	3.47	3.48	3.77	4.88	4.12	3.47	3.32	6.07
48 h	10.8	19.9 *	20.8 *	12.0 *	13.5	24.0 *	23.2 *	12.4	14.2	24.6 *	26.2 *	13.8 *

* *p*-value <0.05 versus BL (paired two-sided Student’s *t*-test). BL, blank; IN, inulin; XA, xanthan; cRG-I, carrot rhamnogalacturonan-I.

## Data Availability

The raw data supporting the conclusions of this article will be made available by the authors on request.

## Supplementary Materials

The following supporting information can be downloaded at https://www.mdpi.com/article/10.3390/microorganisms13081825/s1, Figure S1: The top differentiating species present in the cohort. Figure S2: Relative abundance of *Collinsella intestinalis* per treatment group. Black dots in the figure represent outliers. cRG-I, carrot rhamnogalacturonan-I. Figure S3: Acetate, propionate, and butyrate levels at 48 h in individual donors. (A) Acetate. (B) Propionate. (C) Butyrate. Figure S4: BCFA and ammonium levels at 48 h in small (top row), medium-sized (middle row), and large (bottom row) dogs.
