# Supplementary material for: Effects of cRG-I Prebiotic Treatment on Gut Microbiota Composition and Metabolic Activity in Dogs In Vitro
Source: Microorganisms. 2025 Aug 5;13(8):1825. doi: 10.3390/microorganisms13081825 (PMC12388317; doi:10.3390/microorganisms13081825)

Supplementary Figure S1

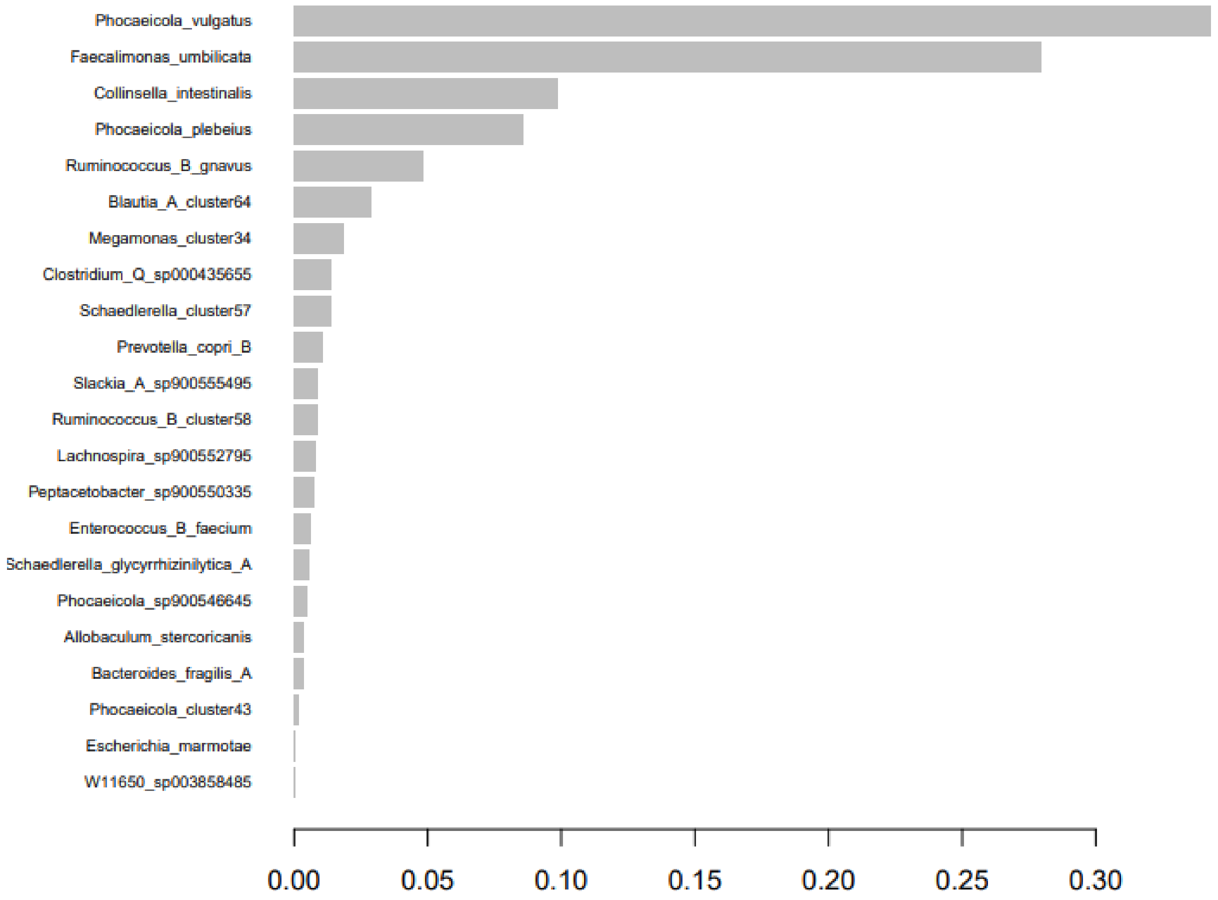

Top differentiating species present in the cohort.

Supplementary Figure S2

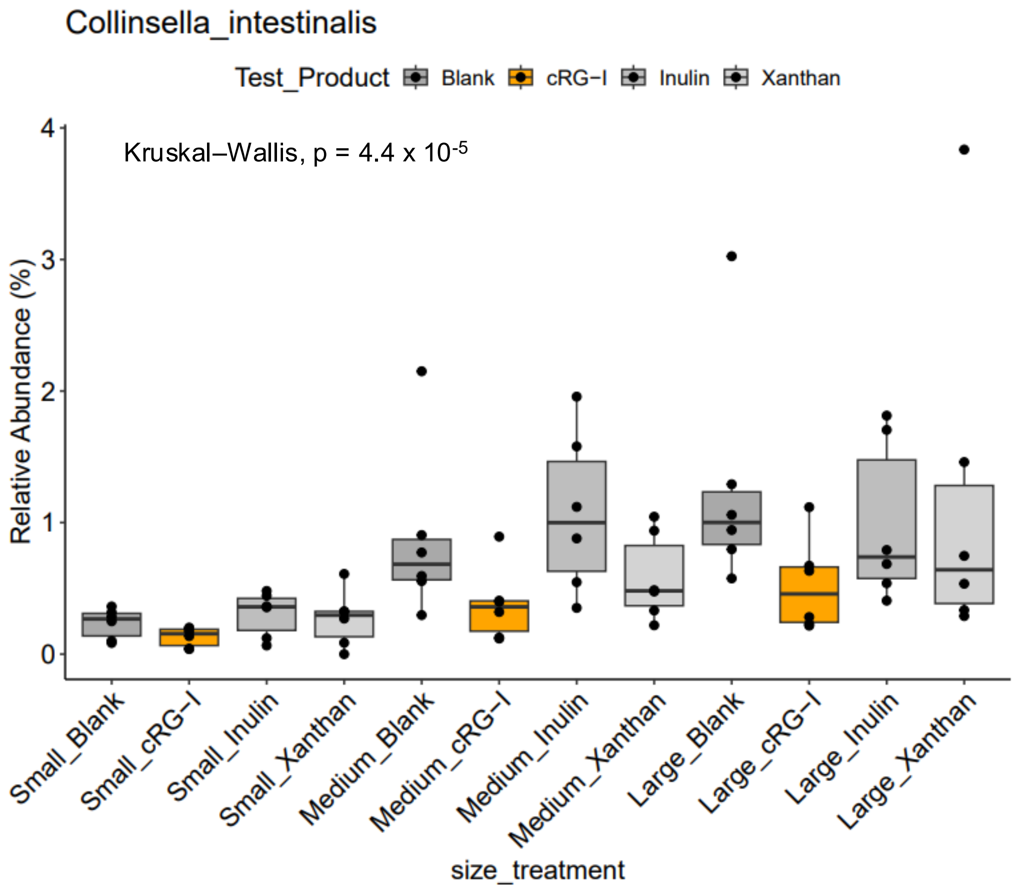

Supplementary Figure S3

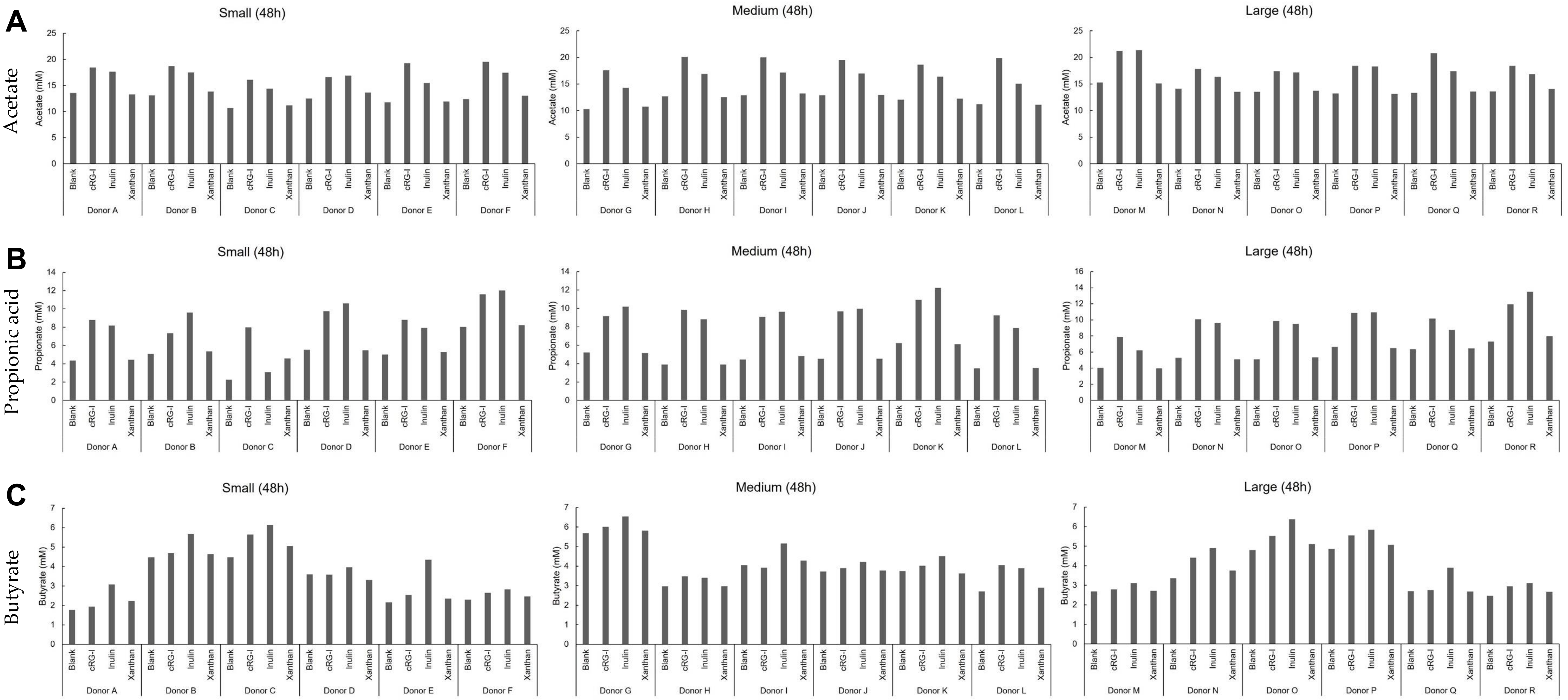

Supplementary Figure S4

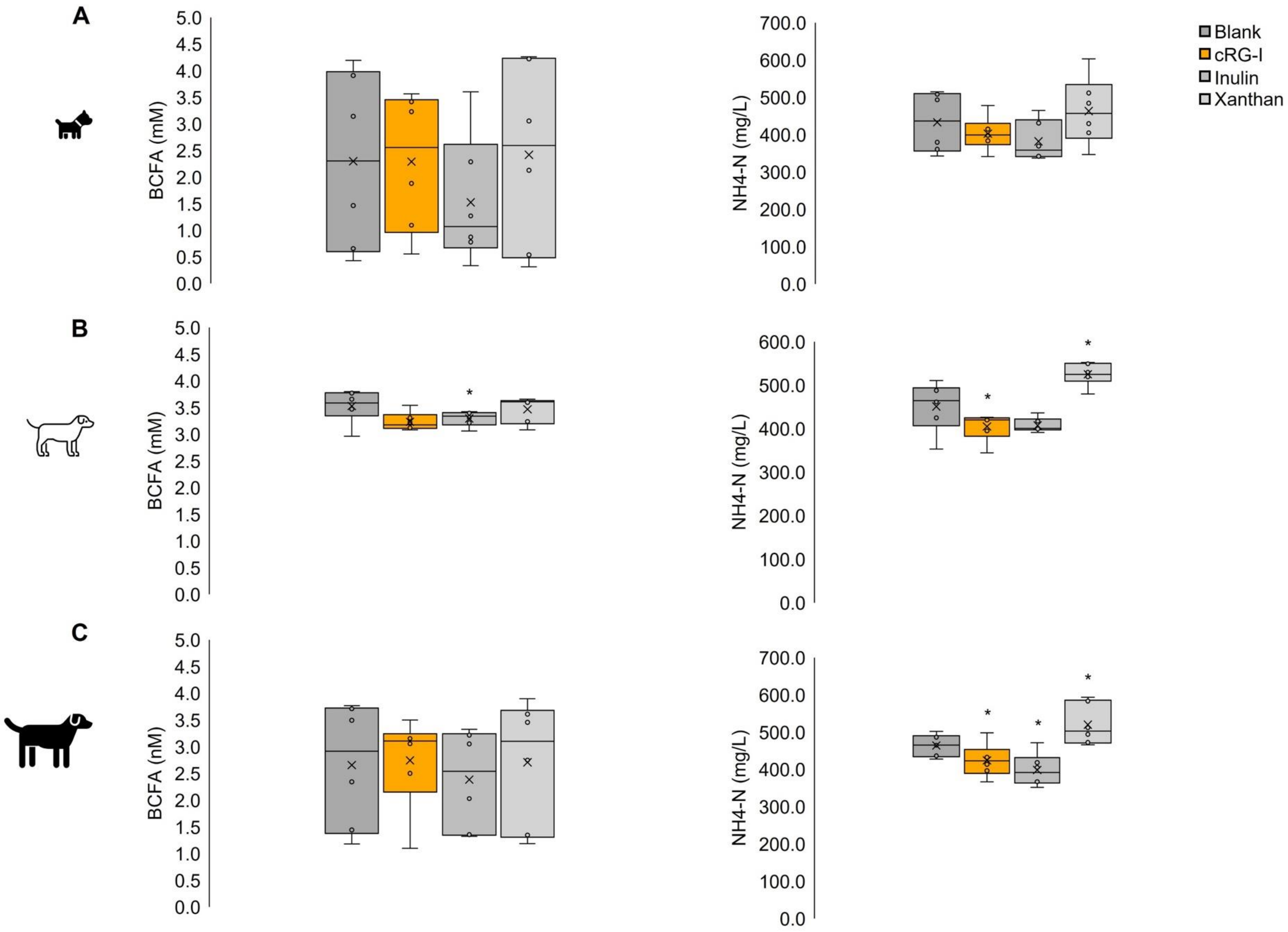

Supplement: Supplementary file 1 [file microorganisms-13-01825-s001.zip › microorganisms-3739259-supplementary.pdf]
